# A Case Report and Literature Review on a Primary Dedifferentiated Liposarcoma of the Gallbladder

**DOI:** 10.7759/cureus.87302

**Published:** 2025-07-04

**Authors:** Victoria Xie, Richard Dominic Luyun, Justin Rivard, Mohamed Akra, Yi Yan, Miao Lu

**Affiliations:** 1 Interdisciplinary Health Program, Rady Faculty of Health Sciences, University of Manitoba, Winnipeg, CAN; 2 Max Rady College of Medicine, University of Manitoba, Winnipeg, CAN; 3 Surgery, Max Rady College of Medicine, University of Manitoba, Winnipeg, CAN; 4 Radiation Oncology, Max Rady College of Medicine, University of Manitoba, Winnipeg, CAN; 5 Radiology, University of Manitoba, St. Boniface General Hospital, Winnipeg, CAN; 6 Pathology, Max Rady College of Medicine, University of Manitoba, Winnipeg, CAN

**Keywords:** dedifferentiated liposarcoma, gallbladder, liposarcoma, mdm2, primary

## Abstract

Primary dedifferentiated liposarcoma (DDLPS) of the gallbladder is an exceptionally rare neoplasm. We present a case of incidentally discovered gallbladder DDLPS identified during routine surveillance abdominal computed tomography (CT) in a patient with a history of low-grade papillary urothelial carcinoma of the bladder. The mass was initially suspected to be primary gallbladder carcinoma, prompting cholecystectomy. Histopathologic examination revealed a high-grade sarcomatous nodule within the gallbladder adventitia, sharply demarcated from an adjacent well-differentiated liposarcoma (WDLPS) component. The diagnosis of DDLPS was confirmed by fluorescence in situ hybridization (FISH) demonstrating MDM2 gene amplification. This case expands the limited literature on gallbladder DDLPS and emphasizes the importance of including liposarcoma in the differential diagnosis of atypical gallbladder masses. Recognition of this rare entity is critical for appropriate diagnosis, surgical decision-making, and patient management.

## Introduction

Dedifferentiated liposarcoma (DDLPS) is a malignant soft tissue neoplasm characterized by a nonlipogenic sarcomatous component adjacent to areas of well-differentiated liposarcoma (WDLPS). It accounts for approximately 20% of all liposarcomas and typically affects middle-aged to older adults, with no significant gender predilection [1-3]. DDLPS most frequently arises in the retroperitoneum, deep soft tissues of the extremities, and paratesticular region. Clinically, it follows an aggressive course, with a local recurrence rate of approximately 40% and a metastatic rate ranging from 15% to 30% [1-3].

Primary DDLPS of the gallbladder is exceedingly rare. Fewer than a dozen cases have been reported in the literature [4-14], with only four confirmed as DDLPS based on histologic features and/or demonstration of MDM2 gene amplification by fluorescence in situ hybridization (FISH) [8,9,11,13]. Owing to their rarity, gallbladder liposarcomas are rarely diagnosed preoperatively. Their nonspecific clinical presentation and imaging findings pose considerable diagnostic challenges. Nevertheless, accurate preoperative diagnosis is essential, as liposarcomas typically require complete surgical resection with negative macroscopic margins [1-3].

Most reported gallbladder liposarcomas present as large, symptomatic abdominal masses. Herein, we describe a rare case of asymptomatic gallbladder DDLPS, incidentally identified during surveillance abdominal computed tomography (CT) in a patient with a history of low-grade papillary urothelial carcinoma of the bladder. This case adds to the limited literature and highlights the importance of considering liposarcoma in the differential diagnosis of gallbladder masses.

## Case presentation

**Figure 1:**
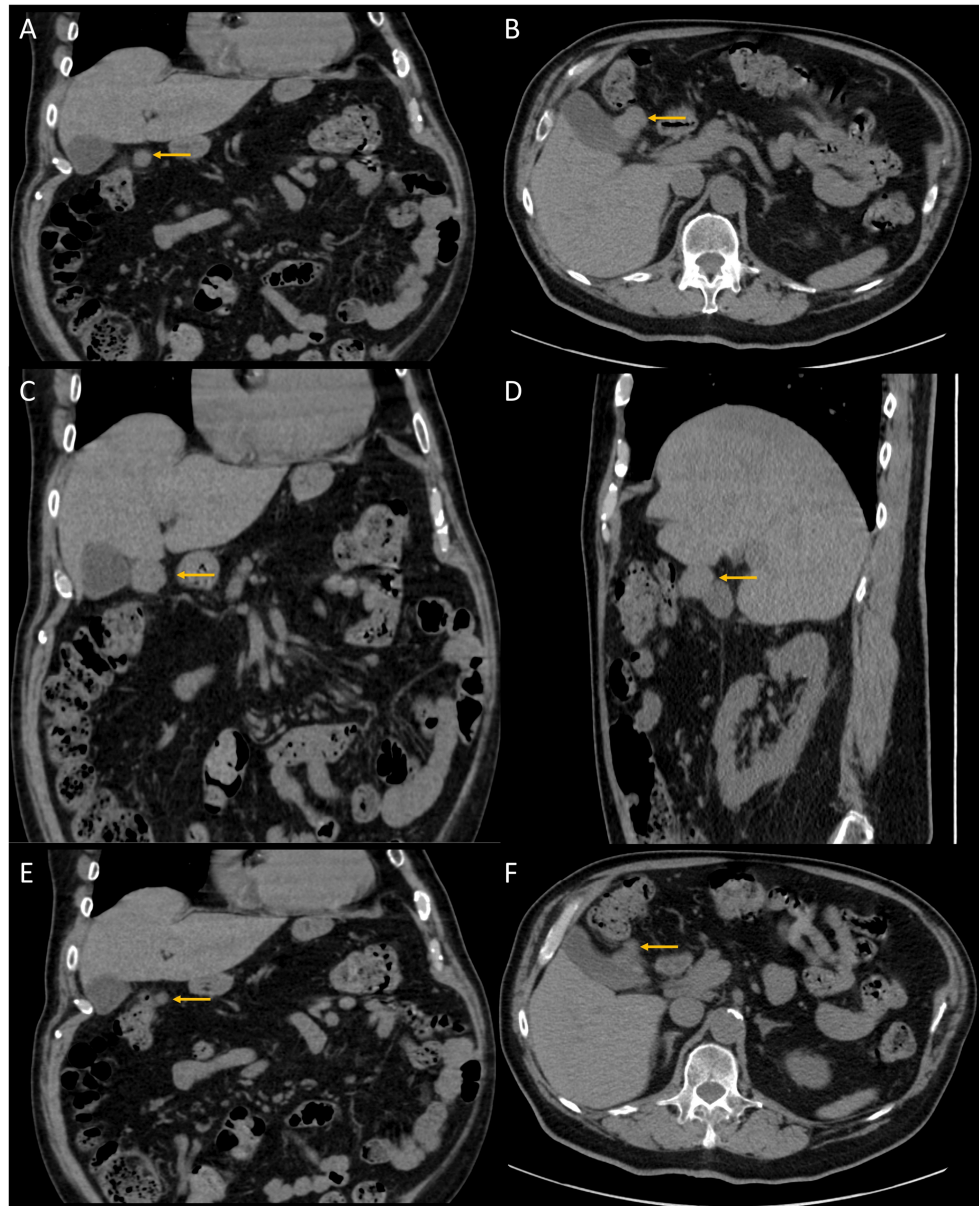
(A-D) Coronal and axial CT images demonstrate a loculated soft tissue mass arising epiphytically from the gallbladder wall and extending into the peritoneum (arrows in A and B). The fat plane between the mass and the inferior margin of the liver is obliterated in both coronal and sagittal views (C and D). (E–F) The mass has increased in size compared to the prior CT scan performed two years ago (arrows)

A 79-year-old man with a history of low-grade noninvasive papillary urothelial carcinoma of the bladder underwent routine surveillance imaging. A contrast-enhanced CT scan of the abdomen and pelvis identified a 3.1 × 2.2 cm exophytic soft tissue mass arising from the gallbladder wall (Figures 1A-1D). A retrospective review of imaging from 2023 revealed that the lesion had previously measured 1.0 × 0.8 cm, indicating interval growth (Figures 1E-1F).

Diffuse mural thickening of the gallbladder was also noted. Based on the imaging findings, the lesion was initially suspected to represent a primary gallbladder carcinoma. The fat plane between the mass and the inferior margin of the liver was obliterated, raising concern for possible hepatic invasion.

The patient was asymptomatic at the time of diagnosis. His past medical history included type 2 diabetes mellitus, hypertension, and transient ischemic attacks. He had no history of prior radiation exposure. Physical examination was unremarkable. Laboratory testing revealed hypoalbuminemia and mild anemia; liver function tests and tumor markers, including carcinoembryonic antigen (CEA) and carbohydrate antigen 19-9 (CA19-9), were within normal limits.

**Figure 2:**
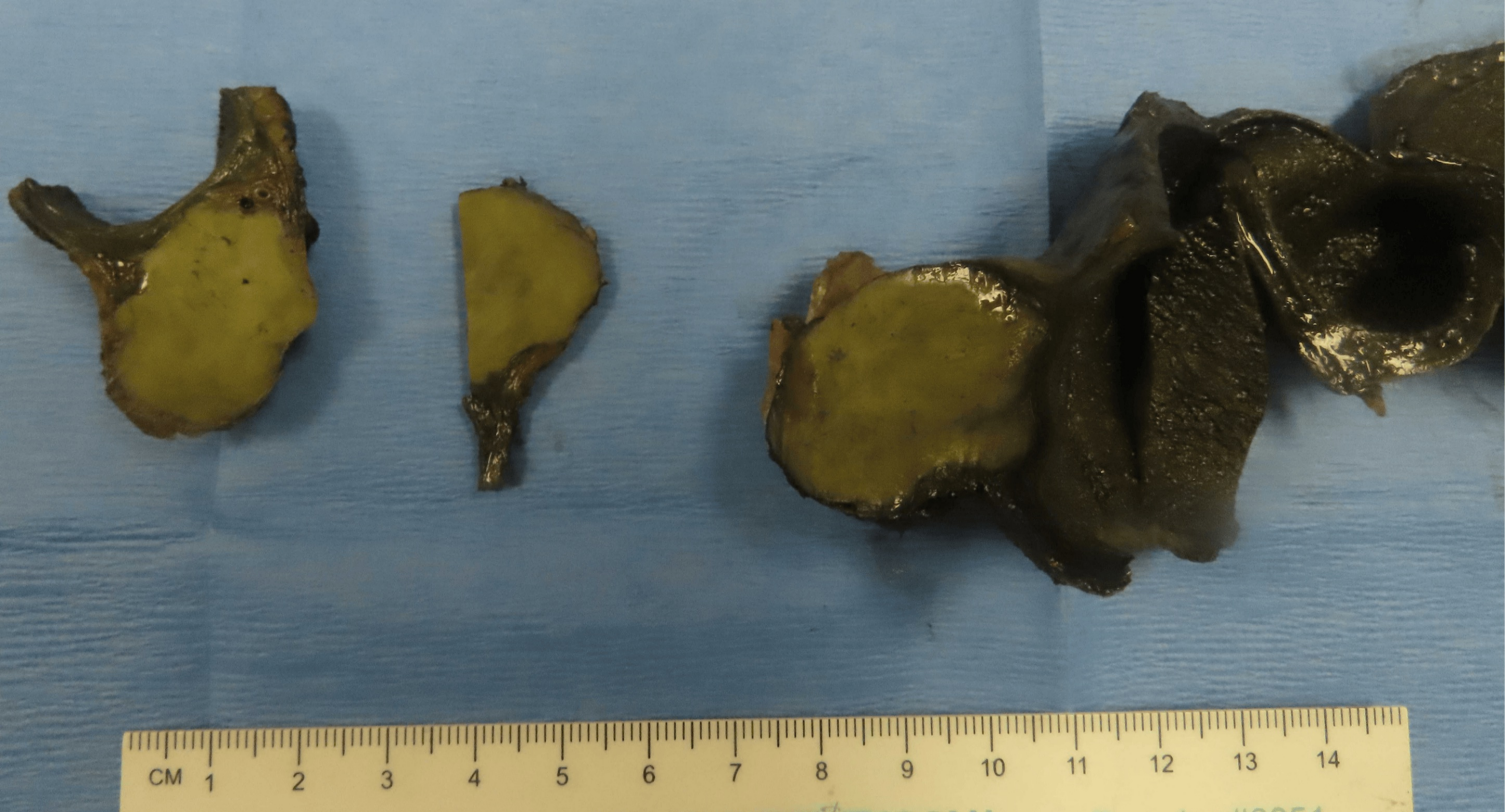
Gross image showing a yellowish-tan, indurated mass located in the adventitia at the gallbladder neck

The patient underwent cholecystectomy with portal lymphadenectomy. Intraoperatively, a firm, well-defined mass was palpated at the neck of the gallbladder without overt invasion into the adjacent liver. Gross examination revealed a gallbladder measuring 9.5 cm in length and 4.7 cm in maximum diameter. A yellowish-tan, indurated mass measuring 3.1 × 2.6 × 2.4 cm was located within the adventitia at the gallbladder neck (Figure 2).

The cut surface of the mass was slightly whorled. The tumor extended to the muscularis propria and abutted the serosal surface but did not reach the hepatic bed or cystic duct margin. The gallbladder wall thickness ranged from 0.2 to 0.5 cm, and the mucosal surface appeared grossly unremarkable. No gallstones were identified.

**Figure 3:**
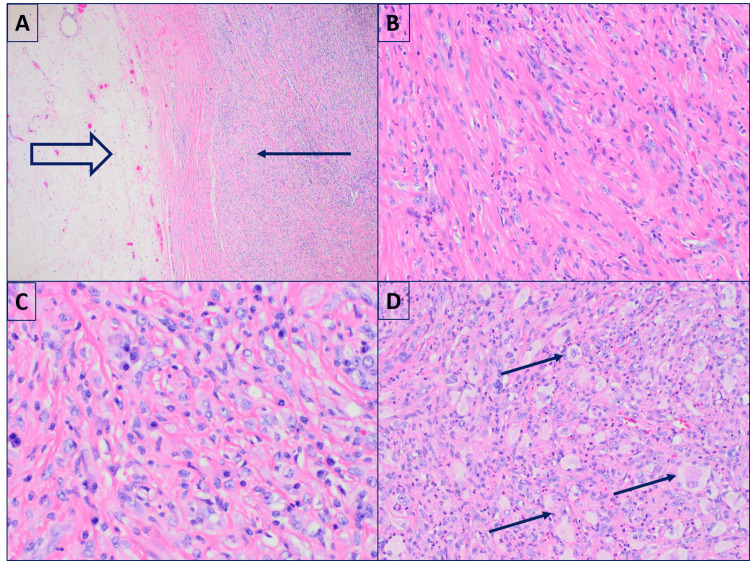
Histologic sections of the nodule reveal a high-grade sarcoma sharply demarcated from the lipomatous component (A) Low-power view (x40) showing separation between the high-grade sarcoma (thin arrow) and the lipomatous component (thick arrow). (B) Spindle cells (x200). (C) Epithelioid/round cells (x200). (D) Histiocyte-like cells (x200), all within a prominent inflammatory stroma

Microscopic examination of the mass revealed a high-grade malignant neoplasm composed of spindle cells, epithelioid cells, and occasional multinucleated tumor cells in a background of prominent inflammation (Figures 3A-3C). Additional foci demonstrated solid sheets of histiocyte-like pleomorphic cells (Figure 3D).

**Figure 4:**
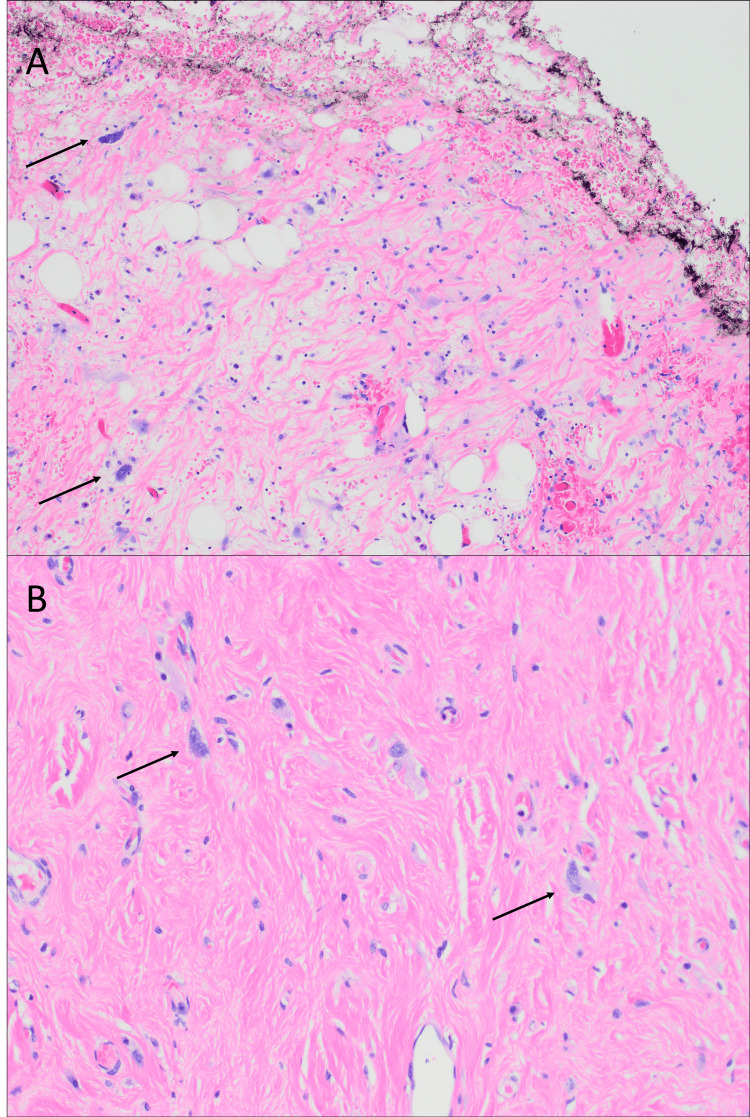
Sections of well-differentiated liposarcoma (WDLPS) (A) Large atypical stromal cells (arrow) in fibrous septa, extending to the inked liver bed resection margin (x200). (B) Sclerosing stroma (x200)

Adjacent sections showed WDLPS involving the adventitia, with atypical hyperchromatic stromal cells in adipose tissue, fibrous septa, and sclerosing stroma accompanied by chronic inflammation (Figures 4A-4B).

Immunohistochemically, the tumor cells were diffusely positive for p16 and negative for pancytokeratin, S100, SOX10, CD34, smooth muscle actin (SMA), desmin, epithelial membrane antigen (EMA), anaplastic lymphoma kinase (ALK), CD68, CD117, and DOG1. FISH testing demonstrated MDM2 gene amplification, supporting a final diagnosis of DDLPS. While the liver bed resection margin was free of high-grade dedifferentiated component, it was involved by the WDLPS component.

The case was discussed at a multidisciplinary tumor board. Given the patient's advanced age, comorbidities, absence of residual high-grade tumor, lack of evidence supporting postoperative radiotherapy, and the potentially high risk of side effects and complications from such treatment, particularly given the low likelihood of meaningful benefit in reducing local recurrence or improving local control, no further surgical or adjuvant therapy was recommended.

## Discussion

Primary liposarcoma of the gallbladder is exceedingly rare, with only 12 cases reported in the literature to date (Table 1) [4-14].

**Table 1:** Summary of the literature of liposarcoma of the gallbladder *: postoperative months/years; **: after the first operation; MLPS: myxoid liposarcoma; PLPS: pleomorphic liposarcoma; DDLPS: dedifferentiated liposarcoma; GB: gallbladder; DOD: died of disease; DOUC: died of unrelated cause; WDLPS: well-differentiated liposarcoma

Year	Author	Age	Sex	Diagnosis	Gross findings	Intervention	Follow-up*
1983	Bader et al. [4]	79	N/A	MLPS	Intramural mass	Cholecystectomy	DOD (2 years)
2006	Hamada et al. [5]	49	F	PLPS	Encapsulated focally; infiltrative 25 x 23 cm	GB and tumor resection	Recurrence (10 and 29 months). Alive (3.5 years)**
2009	Husain et al. [6]	64	F	Liposarcoma	1.7 cm	N/A	DOD
2009	Husain et al. [6]	70	F	Liposarcoma	3.0 cm	N/A	DOUC
2014	Ma et al. [7]	70	F	MLPS	Circumscribed fatty mass 13 x 8 cm	Cholecystectomy	N/A
2018	da Costa et al. [8]	71	F	DDLPS	14 cm	GB and tumor resection, partial hepatectomy	Alive (8 months)
2020	Cheng et al. [9]	83	M	DDLPS	6.0 cm	Cholecystectomy and partial hepatectomy	Alive (4 years)
2021	Ushida et al. [10]	53	M	Metastatic MLPS	4 cm in the submucosa	Laparoscopic cholecystectomy	N/A
2023	Zou et al. [11]	48	F	DDLPS	GB wall mass 5 x 5 x 4.5 cm 2nd mass measuring 7 x 6 x 5 cm near liver capsule	Cholecystectomy and hepatectomy	Alive (2 years)
2024	Chen et al. [12]	32	F	Inflammatory WDLPS	Mass on the body, 5.5 x 5 x 3 cm	Radical resection	Alive (7 months)
2024	Wang et al. [13]	64	F	DDLPS	Mass on the body, 13 x 9 x 7 cm	GB and tumor resection	Alive (15 months)
2025	Yang et al. [14]	35	F	WDLPS	Nodular mass 30 x 24 x 8 cm	GB and mass en bloc resection	N/A

Among these, one patient with gallbladder myxoid liposarcoma (MLPS) had a prior history of a similar tumor in the leg, raising suspicion of metastasis rather than a primary lesion [10]. Most reported cases originated in Asia, with five from China [9,11-14] and two from Japan [5,10]. Patients ranged in age from 32 to 83 years (mean: 60 years), and a strong female predominance was observed (female-to-male ratio of 8:4). The most common presentation was an abdominal mass [5,6,8,9,12-14], while other symptoms included abdominal pain, fever, and dyspepsia. Notably, jaundice was absent in all cases. Laboratory findings were generally nonspecific, although anemia, leukocytosis, elevated C-reactive protein (CRP), and liver enzyme abnormalities were occasionally reported [5,12,14]. Tumor size ranged from 1.7 to 25 cm, with growth patterns variably described as infiltrative [5,8,11,13] or well-circumscribed [7,10,12,14].

Among the 11 confirmed primary gallbladder liposarcomas, four were diagnosed as DDLPS [8,9,11,13], two as WDLPS [12,14], two as MLPS [4,7], one as pleomorphic liposarcoma (PLPS) [5], and two as liposarcoma not otherwise specified [6]. These encompass all four major histologic subtypes recognized by the WHO Classification of Tumours of Soft Tissue and Bone [2]. DDLPS and WDLPS, characterized by MDM2 gene amplification, form a biologic continuum [1,2] and typically arise in the retroperitoneum, extremities, and paratesticular regions, though rare visceral involvement, including the gallbladder, has been documented [15]. In contrast, MLPS and PLPS generally occur in the extremities and are infrequent in the retroperitoneum or intra-abdominal sites [1-3,15].

Radiologically, DDLPS is often suggested by the presence of both soft tissue mass and abnormal fat density or distribution on CT or MRI [16,17]. WDLPS areas may present as heterogeneous fatty regions with thick septa and nodularity, while dedifferentiated regions appear as solid masses with necrosis or hemorrhage [16,17]. In two of the four previously reported gallbladder DDLPS cases, fat-containing regions were observed [8,9]. In the present case, only the dedifferentiated component was evident radiologically and on gross examination, and the WDLPS component was not initially recognized due to the unexpected location.

Grossly, DDLPS typically demonstrates a sharp transition between WDLPS and the nonlipogenic sarcoma, with the latter showing a fleshy, firm, or variegated appearance, occasionally with hemorrhage or necrosis [1-3,15]. Histologically, the WDLPS component is typified by hyperchromatic stromal cells within the fibrous septa of mature adipose tissue. Sclerosing and inflammatory variants are also recognized [1-3,15]. The dedifferentiated component displays high-grade sarcoma morphology with broad heterogeneity, including spindle cell, pleomorphic, and inflammatory MFH-like patterns. Rarely, heterologous elements such as bone, cartilage, or muscle may be present. Homologous lipogenic differentiation, including the presence of lipoblasts, may also be observed. In cases with extensive myxoid change, DDLPS can closely mimic MLPS [1-3,15]. When WDLPS is absent or overlooked, particularly in unusual anatomical sites, diagnosis may be challenging.

Immunohistochemistry can assist, with both WDLPS and DDLPS often showing positivity for MDM2, CDK4, and p16; however, demonstration of MDM2 gene amplification by FISH or polymerase chain reaction (PCR) remains the gold standard for diagnosis [18]. This distinction is critical in differentiating DDLPS from mimics such as MLPS and PLPS. Of note, DDIT3 amplification, observed in one MLPS case, is not specific and may also occur in DDLPS. Only DDIT3 gene rearrangement confirms MLPS [19,20].

Treatment for gallbladder liposarcoma has primarily involved surgical resection. Most patients underwent wide excision, with or without partial hepatectomy. One early case treated with cholecystectomy alone died from widespread recurrence [4]. Another, with incomplete follow-up data, also underwent cholecystectomy only [7]. While one patient who received chemotherapy died of the disease [6] and one experienced multiple recurrences [5], most remained disease-free over follow-up periods ranging from seven months to four years. None received adjuvant radiation therapy.

In our case, liposarcoma was not included in the preoperative differential, and resection was limited to the CT-detected mass. Histologic examination revealed high-grade sarcoma and additional sampling disclosed adjacent WDLPS infiltrating the gallbladder adventitia, resulting in a positive surgical margin at the liver bed.

## Conclusions

Given the rarity of gallbladder DDLPS, accurate diagnosis can be challenging, especially when the tumor arises in an unexpected location or when only the high-grade component is initially evident. This case highlights the importance of thorough radiologic assessment, with special attention to areas of abnormal fat, as well as detailed gross and histologic examination. Extensive sampling and the use of ancillary molecular techniques, particularly FISH for MDM2 amplification, are critical for establishing the correct diagnosis. A multidisciplinary approach involving radiology, surgery, pathology, and oncology is essential for optimal management. Due to the limited number of reported cases, the long-term prognosis remains uncertain, emphasizing the need for careful postoperative surveillance and reporting of additional cases to inform clinical practice.
